# Colocalization of Cocaine- and Amphetamine-Regulated Transcript with Kisspeptin and Neurokinin B in the Human Infundibular Region

**DOI:** 10.1371/journal.pone.0103977

**Published:** 2014-08-01

**Authors:** Katalin Skrapits, Beáta Á. Borsay, László Herczeg, Philippe Ciofi, Stephen R. Bloom, Mohammad A. Ghatei, Waljit S. Dhillo, Zsolt Liposits, Erik Hrabovszky

**Affiliations:** 1 Laboratory of Endocrine Neurobiology, Institute of Experimental Medicine, Hungarian Academy of Sciences, Budapest, Hungary; 2 Department of Forensic Medicine, Faculty of Medicine of the University of Debrecen, Debrecen, Hungary; 3 INSERM U862, Neurocentre Magendie, Bordeaux, France; 4 Department of Investigative Medicine, Hammersmith Hospital, Imperial College London, London, United Kingdom; 5 Department of Neuroscience, Faculty of Information Technology, Pázmány Péter Catholic University, Budapest, Hungary; John Hopkins University School of Medicine, United States of America

## Abstract

Kisspeptin (KP)- and neurokinin B (NKB)- synthesizing neurons of the hypothalamic arcuate nucleus play a pivotal role in the regulation of pulsatile gonadotropin-releasing hormone (GnRH) secretion. Unlike in rodents and sheep, the homologous KP and NKB neurons in the human infundibular region rarely express dynorphin- but often exhibit Substance P (SP) immunoreactivity, indicating remarkable species differences in the neurochemical phenotype of these neurons. In search for additional neuropeptides in human KP and NKB neurons, we carried out immunofluorescent studies on hypothalamic sections obtained from five postmenopausal women. Colocalization experiments provided evidence for the presence of cocaine- and amphetamine-regulated transcript (CART) in 47.9±6.6% of KP-immunoreactive (IR) and 30.0±4.9% of NKB-IR perikarya and in 17.0±2.3% of KP-IR and 6.2±2.0% of NKB-IR axon varicosities. All three neuropeptides were present in 33.3±4.9% of KP-IR and 28.2±4.6% of NKB-IR somata, respectively, whereas triple-labeling showed lower incidences in KP-IR (14.3±1.8%) and NKB-IR (5.9±2.0%) axon varicosities. CART-IR KP and NKB neurons established contacts with other peptidergic cells, including GnRH-IR neurons and also sent projections to the infundibular stalk. KP and NKB fibers with CART often contained SP as well, while being distinct from CART fibers co-containing the orexigenic peptide agouti-related protein. Presence of CART in human, but not rodent, KP and NKB neurons represents a new example of species differences in the neuropeptide repertoire of mediobasal hypothalamic KP and NKB neurons. Target cells, receptor sites and physiological significance of CART in the efferent communication of KP and NKB neurons in primates require clarification.

## Introduction

Peptidergic neurons co-synthesizing kisspeptin (KP), neurokinin B (NKB) and dynorphin in the hypothalamic arcuate nucleus (ARC) [1]–[3] (‘KNDy neurons’ [4]) play a pivotal role in negative sex steroid feedback to the hypophysiotropic gonadotropin-releasing hormone (GnRH) neurons [1], [5], [6]; in rats, neurotoxic ablation of these cells prevents the rise in serum LH and attenuates the rise in serum FSH following ovariectomy [7]. The network connectivity and peptidergic communication of KNDy neurons with each other and with GnRH neurons have also been implicated in the regulation of the GnRH/LH pulse generator [2], [3], [8]; while NKB increases, dynorphin decreases the frequency of LH secretory episodes in gonadectomized goats [3].

NKB and dynorphin, derived at least partly from intrinsic sources, can act on KNDy neurons via autoreceptors. Such autoreceptors include the NK3 [2], [9], [10], NK1 [11] and NK2 [11] tachykinin receptors through which NKB can excite [11]–[13], and the κ-opioid autoreceptor through which dynorphin and selective kappa-opioid receptor agonists can inhibit [11], [13] KNDy neuronal activity. In contrast with NKB and dynorphin, intrinsic KP does not seem to influence the electric activity of KNDy neurons [11], but rather, plays an important role in the neuronal transmission from KNDy to GnRH neurons, the latter expressing KISS1R [14]–[16]. Indeed, the pulsatile KP output into the median eminence of the female rhesus monkey is temporally correlated with GnRH neurosecretory pulses [17].

The distribution and afferent regulation of GnRH neurons can largely differ in humans and laboratory species, making the relatively rare neuroanatomical observations on humans particularly valuable [18]. Recent immunohistochemical studies in our laboratory have established that the neurochemical characteristics of mediobasal hypothalamic KP and NKB neurons in the human are often different from those reported from laboratory species. Accordingly, unlike in rodents, KP and NKB neurons of the human infundibular region rarely display dynorphin- [19] but often exhibit Substance P (SP) [20] immunoreactivity. Furthermore, the extent of colocalization between KP and NKB is far from being complete, which is particularly visible in young men [19].

Cocaine- and amphetamine-regulated transcript (CART) is a potent stimulator of both GnRH and KP neurons, as established recently in rats and mice [21]. In these rodent species, a major source of CART in neuronal afferents to GnRH and KP cells is the ARC [21] where CART is colocalized with the proopiomelanocortin (POMC)-derived anorexigenic peptide α-MSH [22]. The anatomical distribution of CART in the ARC is surprisingly different in primates whose α-MSH neurons do not contain CART [23]. Instead, a large subset of CART neurons in the infundibular (arcuate) nucleus (Inf) of the human actually corresponds to putative orexigenic neurons which co-express neuropeptide Y (NPY) with agouti-related protein (AGRP) [24]. In the present study we have addressed the possibility that the remaining ∼46% of CART neurons in the human Inf are identical, at least partly, with KP and/or NKB neurons. To provide evidence for this hypothesis, we have carried out a series of immunofluorescent experiments on histological specimens from human hypothalami. *Post mortem* tissue samples were used from postmenopausal women, a human model containing the highest levels of KP, NKB and SP immunoreactivities in the Inf [20], [25], [26]. First, the extent of colocalization between KP, NKB and CART immunoreactivities in neuronal cell bodies as well as fibers of the Inf was assessed quantitatively in triple-labeled specimens. Next, we addressed whether CART-IR KP and NKB fibers are identical with those coexpressing SP with KP and NKB [20] and/or with orexigenic neurons coexpressing AGRP with CART [24]. Finally, the connectivity of CART-containing KP and NKB fibers to various peptidergic cell types of the infundibular region, including GnRH cells, was analyzed.

## Materials and Methods

### Ethics statement

Permission to carry out tissue collection and experiments was obtained from the Regional Committee of Science and Research Ethics (DEOEC RKEB/IKEB: 3183-2010) and according to the Hungarian Law (1997 CLIV and 18/1998/XII.27. EÜM Decree). Authors of this manuscript collected human hypothalamic tissue samples during autopsies at the Forensic Medicine Department of the University of Debrecen. Patient information was initially available and in all subsequent studies, the subjects were anonymized.

### Human subjects

Hypothalamic tissue blocks from five postmenopausal women (64–90 years) were used. Selection criteria included lack of history of neurological and endocrine disorders and *post mortem* intervals below 48 h. Tissue collection, section preparation and sample processing for immunohistochemistry were carried out as in earlier immunohistochemical studies on human KP and NKB neurons [19], [20], [25], [27], [28].

### Section preparation for immunohistochemistry

The hypothalamic tissue blocks were first rinsed briefly with running tap water and then, immersion- fixed with 4% formaldehyde in 0.1 M phosphate buffer saline (PBS; pH 7.4) for 7–14 days. The fixed hypothalami were trimmed in a way to include the optic chiasm rostrally, the mammillary bodies caudally and the anterior commissure dorsally [19], [20], [25], [27], [28]. Sagittal cuts were made 2 cm lateral to the midsagittal plane on both sides. Subsequently, the blocks were cut in halves and infiltrated with 20% sucrose for 5 days at 4°C. The right hemihypothalami were placed in a freezing mold, surrounded with Jung tissue freezing medium (Leica Microsystems, Nussloch Gmbh, Germany; diluted 1∶1 with 0.9% sodium chloride solution), snap-frozen on powdered dry ice, and stored at −80°C until sectioned at 30 µm in the coronal plane with a Leica SM 2000R freezing microtome (Leica Microsystems). The sections were stored permanently in anti-freeze solution (30% ethylene glycol; 25% glycerol; 0.05 M phosphate buffer; pH 7.4) at −20°C.

### Tissue pretreatments

Every 72^nd^ section of the Inf (2–4 sections per subject) was processed for each immunofluorescent experiment. The sections were rinsed copiously in PBS and pretreated with a mixture of 0.5% H_2_O_2_ and 0.2% Triton X-100 for 30 min. Then, epitope retrieval was carried out using 0.1 M citrate buffer (pH 6.0) at 80°C for 30 min. To reduce non-specific antibody binding, the sections were incubated in 2% normal horse serum (NHS) in PBS for 20 min.

### Section mounting and coverslipping

The sections were processed for five different immunofluorescent experiments as detailed below, then, mounted on microscope slides from 0.1 M Tris-HCl buffer (pH 7.6) and coverslipped with the aqueous mounting medium Mowiol.

### Confocal microscopy

Confocal images were prepared with a Radiance 2100 confocal system (Bio-Rad Laboratories, Hemel Hempstead UK). Two-µm-thick single optical slices were collected for illustrations and analyses. Four different fluorochromes were used and detected with the following laser lines: 405 nm for AMCA, 488 nm for FITC, 543 nm for Cy3, 637 nm for Cy5. Dichroic/emission filters were as follows: 500 nm/420–480 nm for AMCA, 560 nm/500–540 nm for FITC, 650 nm/560–610 nm for Cy3 and 660-nm-long pass filter for Cy5. Sequential line scanning (lambda strobing function) was used so that only one excitation laser and the corresponding emission detector were active during a line scan, to eliminate any emission crosstalk between the fluorophores. Single-labeled control sections included in each experiment were used as further negative controls to exclude bleed-through. The separately recorded green, red and far-red channels were merged and transferred into the green, red and blue channels of Adobe Photoshop (PSD) files, respectively, in RGB mode. The fourth channel used to visualize AMCA in quadruple-labeling studies was either handled as a separate black-and-white image (experiment 3) or illustrated in the same image with a brown pseudocolor (experiment 5).

### Experimental design

#### Experiment 1. Triple-immunofluorescent studies to localize CART in KP-IR and NKB-IR perikarya of the mediobasal hypothalamus

The sections were incubated in a cocktail of the following three primary antibodies raised in different species. i) The GQ2 sheep KP antiserum (1∶1000) has been generated against human KP-54 [29]. It recognizes human KP-54, KP-14 and KP-10, while showing virtually no cross-reactivity (<0.01%) with other related human RF-amide peptide, including prolactin releasing peptide, neuropeptide FF, neuropeptide AF and RF-amide related peptides (RFRP1, RFRP2, RFRP3) [29]. The GQ2 antiserum has been used successfully in previous immunohistochemical experiments to detect KP neurons of the rhesus monkey [30], [31] and the human [19], [20], [25], [27], [28]. ii) The detection of NKB synthesizing neurons used a rabbit polyclonal antiserum against the C-terminal 28 amino acids of human pro-NKB (IS-682; P. Ciofi; 1∶1000) [19], [20], [25], [27], [28], [31]. iii) The CART-IR neuronal elements were detected with a mouse monoclonal CART antibody (CA61F4OP001, 2 µg/mL; gift from Dr. Jes Thorn Clausen, Novo Nordisk A/S, Denmark) [32]. Incubation in the primary antibody mixture for 48 h at 4°C was followed by a cocktail of secondary antibody-fluorochrome conjugates for 5 h at room temperature: anti-sheep-Cy3, 1∶1000; anti-mouse-FITC, 1∶250; anti-rabbit-Cy5, 1∶500 (ea. from Jackson ImmunoResearch Laboratories, West Grove, PA, USA). The colocalization of immunoreactivities in cell bodies was illustrated in 2-µm-thick single optical slices.

The 2-µm-thick optical slices were also analyzed to obtain a quantitative estimate about the extent of CART coexpression in KP-IR and NKB-IR neuronal cell bodies. Representative photomicrographs (1–3/human subject) were taken to include the highest number of KP and NKB neurons from the Inf. Results were expressed as the mean percentages±SEM of the five individuals and presented graphically in a pie diagram where 100% corresponded to the total number of the detectable (KP and/or NKB)-IR neurons in each subject (N = 515 in the five individuals). The incidence of single-labeled CART neurons, many of which could be identical with AGRP/NPY cells in other subregions of the Inf [24], was not illustrated in the chart, in view that these neurons were likely underrepresented in the photographic samples centered on KP and NKB cell groups. Additionally, column graphs were used to illustrate the relative incidences of KP-IR and NKB-IR neurons exhibiting single-, double- or triple-neuropeptide phenotypes.

#### Experiment 2. Visualization of KP-IR and NKB-IR fiber varicosities with and without CART labeling

Adobe Photoshop files containing the 2-µm-thick optical slices were also used to study the CART-IR KP and NKB fiber varicosities and their connectivity to the infundibular stalk (InfS). Quantitative studies were carried out to determine the percentages of KP-IR and NKB-IR axon varicosities that co-contained CART in areas where KP and NKB neurons occurred at the highest densities. A total section area of ∼700,000 µm^2^ was sampled from a 2-µm-thick single optical slice of each human individual. First, a grid with 20×20 µm squares was superimposed on the photographs. Then, axon varicosities that overlapped with the gridlines in any color channel were analyzed and categorized based on their neuropeptide signal composition. The optical channels were switched on and off during the analysis when necessary and areas containing labeled cell bodies or artifacts were omitted from the study. The relative incidences were expressed as the mean percentages±SEM of all (KP and/or NKB)-IR varicosities/11270 (KP and/or NKB)-IR varicosities from the five individuals/. The single-labeled CART-IR axon varicosities were not illustrated in this diagram for the sake of consistency with the colocalization data in cell bodies. The relative incidences of KP-IR and NKB-IR axons with single-, double- or triple-neuropeptide phenotypes were illustrated in column graphs.

#### Experiment 3. Triple-immunofluorescent studies to determine whether CART-IR NKB neurons and CART-IR AGRP neurons are identical or distinct

The rabbit NKB antiserum (1∶1000), the mouse CART antibody (2 µg/mL) and a guinea pig AGRP antiserum (GP-029-50; 1∶1000; Biosensis Pty. Ltd., Temecula, CA, USA) were used in a cocktail for 48 h at 4°C and then, reacted with the mixture of anti-rabbit-Cy3 (1∶1000), anti-mouse-FITC (1∶250) and anti-guinea pig-Cy5 (1∶500) conjugates (Jackson ImmunoResearch Laboratories) for 5 h at room temperature. The three fluorochromes were detected and visualized in separate channels of Adobe Photoshop files, as described in experiments 1 and 2. Using a similar sampling approach as in experiment 2, the phenotype of labeled axon varicosities crossing gridlines was analyzed in digital photomicrographs over a 240,000 µm^2^ area from each of 3 individuals.

#### Experiment 4. Quadruple-immunofluorescent studies to simultaneously detect KP, NKB, CART and SP in the same neuronal fibers

To determine whether the CART-IR and the SP-IR subpopulations of KP and NKB neurons are identical or different, the primary antibody cocktail used in experiments 1 and 2 was supplemented with a rat monoclonal SP antibody (Serotec #8450-0505; Bio-Rad Laboratories, Inc., Hercules, CA; 1∶3,000) [33]. Following a 48-h-incubation in this mixture at 4°C, a cocktail of four different secondary antibody-fluorochrome conjugates were applied to the sections for 5 h at room temperature: anti-rat-AMCA, 1∶50; anti-mouse-FITC, 1∶250; anti-sheep-Cy3, 1∶1000; anti-rabbit-Cy5, 1∶500 (ea. from Jackson ImmunoResearch Laboratories). The visualization of the first three neuropeptides (KP, NKB and CART) was carried out as in experiments 1 and 2, whereas the SP signal was illustrated separately in a parallel black-and-white image. The presence/absence of quadruple-labeled neuronal elements was determined by switching between the superimposed Photoshop layers.

#### Experiment 5. Studies to analyze the connectivity of CART-IR KP and NKB fibers

Sections from experiments 1, and 4 were analyzed at high power to study the neuronal connectivity of CART-IR neurons. In an additional quadruple-labeling study, the primary antibody cocktail from experiment 1 was supplemented with a polyclonal guinea pig GnRH antiserum (#1018; 1∶3000) [25]. This was followed by the cocktail of secondary antibody-fluorochrome conjugates: anti-guinea pig-AMCA, 1∶50; anti-mouse-FITC, 1∶250; anti-sheep-Cy3, 1∶1000; anti-rabbit-Cy5, 1∶500 (Jackson ImmunoResearch Laboratories) for 5 h. The visualization of KP, NKB and CART was carried out as in experiments 1 and 2. The AMCA signal for GnRH was overlaid to this three-color image in a separate Adobe Photoshop layer (screen mode), after being converted to a brown pseudocolor.

### Specificity controls

All secondary antibodies were raised in donkeys and recommended for multiple labeling by Jackson ImmunoResearch Laboratories. The lack of their cross-reactivity was confirmed by omitting one of the primary antibodies from the multiple-labeling experiments.

Multiple controls were combined to rule out the possibility of labeling artifacts due to cross-reactivity of the primary antibodies. To provide controls for the labeling specificity of CART, test sections were incubated overnight in primary antibody solution preabsorbed overnight with 10^−4^ M human CART 54–102 and then, processed further for the immunofluorescent detection. As a positive control, the immunohistochemical detection of CART in KP neurons has been replicated with the use of a rabbit polyclonal reference CART antiserum [34] (1∶1000; gift from Dr. M.J. Kuhar). As described elsewhere, specificity tests for GnRH [25], KP [28], NKB [28] and SP [20] labeling included the comparative analysis of labeling patterns obtained with two distinct antisera. Similar experiments were carried out here to test for the specificity of CART labeling with the rabbit CART antiserum and AGRP labeling with affinity-purified goat AGRP antibodies from a different resource (Everest Biotech Ltd., Oxfordshire, UK). In colocalization experiments, the existence of many bright single-labeled, in addition to double-, triple- and quadruple-labeled neuronal structures served as endogenous controls for the absence of antibody cross-reactions.

In addition to using sequential line scanning which eliminated any emission crosstalk between the fluorophores, single-labeled test specimens were included in each study to confirm that each chromogen provides signal in a single detector only.

## Results

The distribution of CART-IR elements in the human hypothalamus was in agreement with previous findings [35]. Accordingly, labeled neurons were present in the lateral hypothalamic area, the posterior hypothalamus and in the paraventricular, supraoptic, periventricular and infundibular nuclei [35].

### Experiment 1. KP-IR and NKB-IR perikarya often exhibit CART co-labeling

**Figure 1:**
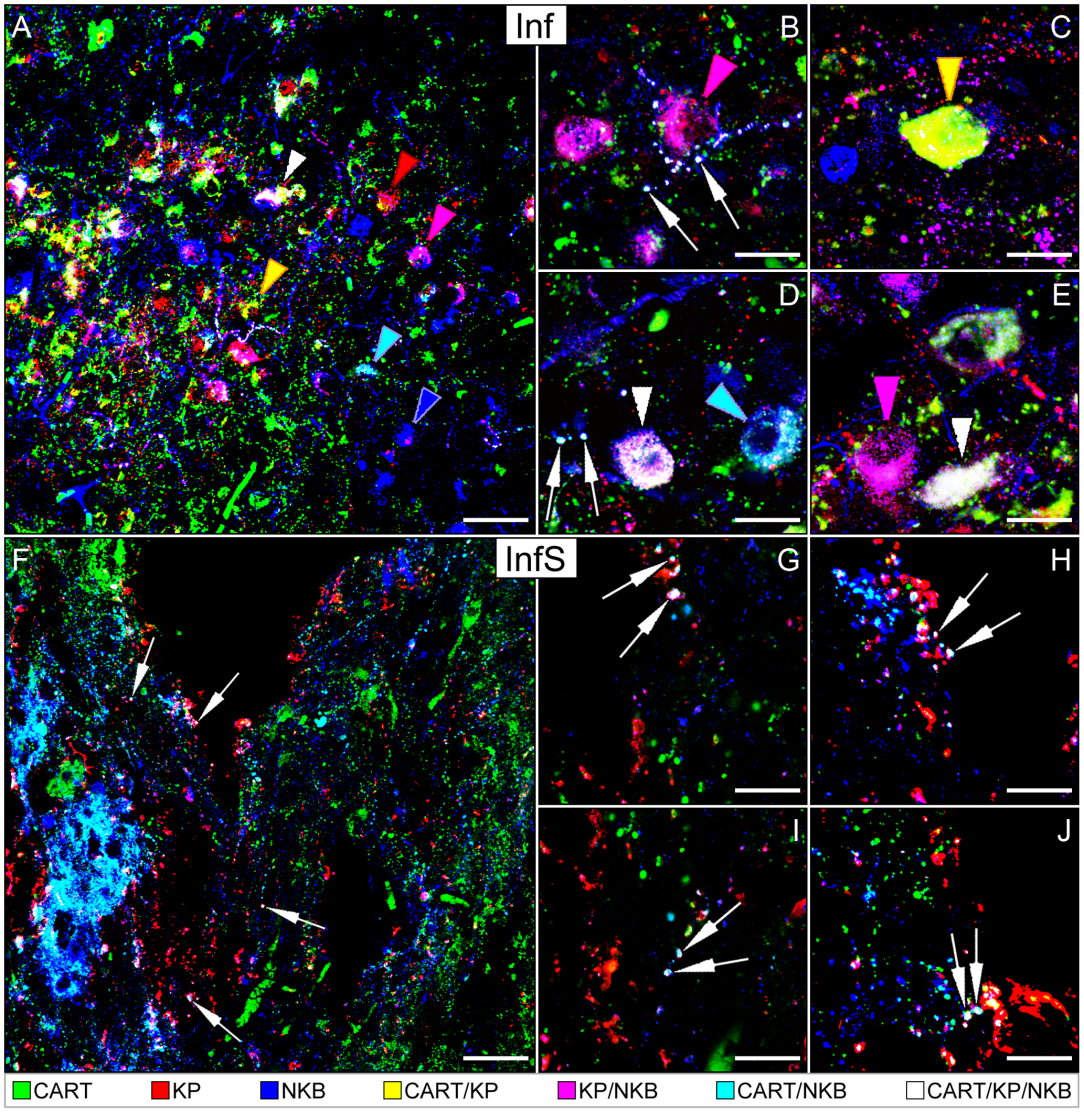
Immunofluorescent localization of cocaine- and amphetamine-regulated transcript (CART) in kisspeptin (KP) and neurokinin B (NKB)-immunoreactive (IR) neuronal elements of the infundibular region. Triple-label immunofluorescent detection of CART (green), KP (red) and NKB (blue) immunoreactivities in the infundibular nucleus (Inf; **A–E**) and stalk (InfS; **F–J**) of postmenopausal women reveals a substantial degree of colocalization between the three neuropeptides. Examples for single-, double- and triple-labeled perikarya are indicated by color-coded arrowheads, whereas white arrows point to triple-labeled axon varicosities. For color-coding, see captions at the bottom of the figure. Low-power confocal image in **A** illustrates that considerable subsets of KP-IR (47.9±6.6%) and NKB-IR (30.0±4.9%) cell bodies in the Inf also exhibit CART signal; many of these cells are triple-labeled (white arrowhead). High-power insets show examples for different colocalization patterns. The KP and NKB neurons of the Inf, with or without CART labeling, also send abundant projections to the InfS (**F–J**). CART in the InfS may act on neurosecretory terminals to regulate reproduction via autocrine/paracrine mechanisms. In addition, it may also enter the portal circulation to act in the adenohypophysis. Scale bars = 50 µm in **A**, 60 µm in **F** and 20 µm in **B–E** and **G–J**.

CART-IR neuronal cell bodies were often detectable in subregions of the Inf that were also populated by KP-IR and NKB-IR neurons (Fig. 1A). The analysis of confocal images provided evidence for the presence of CART signal in subsets of KP-IR and NKB-IR neuronal cell bodies and fibers (Figs. 1A–E).

**Figure 2:**
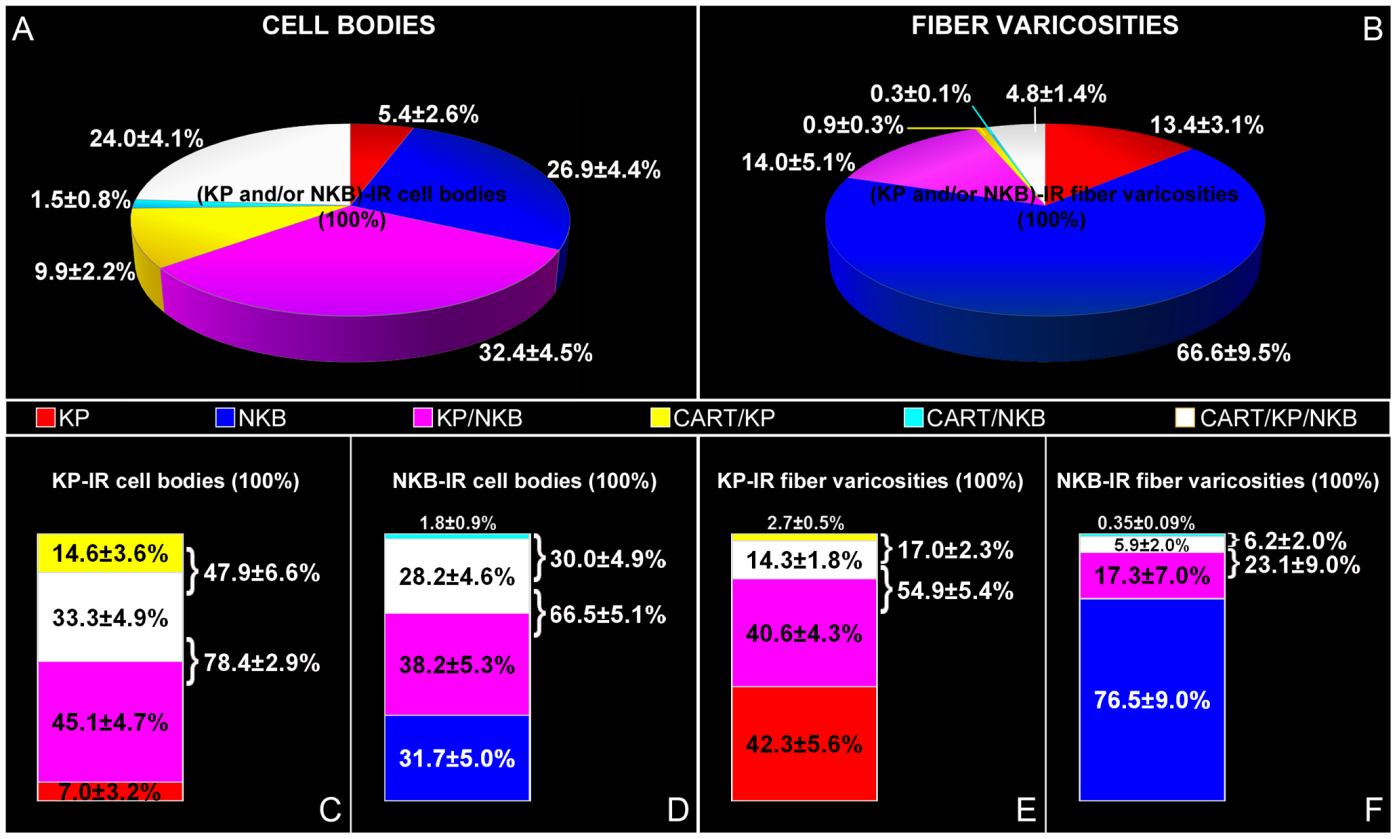
Incidences of KP-, NKB- and CART-IR perikarya and axon varicosities in the Inf. Pie charts in **A** and **B** illustrate the relative incidences of different labeling patterns, expressed as percentages of the total number of (KP and/or NKB)-IR neuronal elements (cell bodies in **A** and fiber varicosities in **B**; %±SEM of five postmenopausal women). Note in **A** that the three most frequently encountered perikaryon phenotypes, in order, co-contains KP and NKB and is devoid of CART [purple color; 32.4±4.5% of all (KP and/or NKB)-IR neurons], contains NKB signal only [blue color; 26.9±4.4% of all (KP and/or NKB)-IR neurons] or contains NKB, KP and CART together [white color; 24.0±4.1% of all (KP and/or NKB)-IR neurons]. Chart in **B** illustrates the mean incidences of the distinct types of axon varicosities, out of which single-labeled NKB fibers represent the most frequently encountered phenotype. Note that, in general, neuropeptide signals are coexpressed less frequently in axons than in cell bodies (compare **B** to **A**). Column graphs in **C–F** illustrate the percentages of KP-IR (**C**, **E**) and NKB-IR (**D**, **F**) neuronal elements (cell bodies in **C–D** and fiber varicosities in **E–F**) that are single-, double-, and triple-labeled. As shown in **C**, 78.4±2.9% of KP-IR cell bodies also contain NKB, 47.9±6.6% contain CART and 33.3±4.9% are triple-labeled. On the other hand, 66.5±5.1% of NKB-IR perikarya also contain KP, 30.0±4.9%, contain CART and 28.2±4.6% are triple-labeled (**D**). Regarding KP-IR fiber varicosities, 54.9±5.4% contain NKB, 17.0±2.3% contain CART and 14.3±1.8% are triple-labeled (**E**), whereas out of all NKB-IR axon varicosities, 23.1±9.0% contain KP, 6.2±2.0% contain CART signal and 5.9±2.0% are triple-labeled for KP/CART/NKB (**F**).

The quantitative analysis of 515 (KP and/or NKB)-IR somata from five individuals established that the three most frequently encountered neuronal phenotypes contained KP and NKB without CART (32.4±4.5%), NKB signal only (26.9±4.4%) and NKB, KP and CART together (24.0±4.1%) (see pie diagram in Fig. 2A).

Of all KP-IR perikarya, 78.4±2.9% contained NKB, 47.9±6.6% contained CART and 33.3±4.9% were triple-labeled for KP/NKB/CART (Fig. 2C). On the other hand, 66.5±5.1% of NKB-IR perikarya contained KP, 30.0±4.9% contained CART and 28.2±4.6% were triple-labeled (Fig. 2D).

### Experiment 2. Smaller subsets of KP-IR and NKB-IR fiber varicosities in the Inf and the InfS exhibit CART co-labeling

As also noticed in earlier studies [25], [27], [28], the colocalization between KP and NKB was relatively infrequent in axons, compared with cell bodies (Compare pie chart in Fig. 2A to B). Similarly, we found that the ratios of CART-expressing KP and NKB fibers were low (Fig. 2B), compared with the incidences of CART-expressing KP and NKB perikarya (Fig. 2A).

The quantitative analysis of 11270 (KP and/or NKB)-IR axon varicosities from five individuals revealed that the most frequently encountered fiber phenotypes contained NKB only (66.6±9.5%), both KP and NKB without CART (14.0±5.1%) and KP only (13.4±3.1%) (see pie chart in Fig. 2B).

Of all KP-IR axon varicosities, 54.9±5.4% contained NKB, 17.0±2.3% contained CART and 14.3±1.8% were triple-labeled (Fig. 2E). Of all NKB-IR axon varicosities, 23.1±9.0% contained KP and 6.2±2.0% contained CART, 5.9±2.0% being triple-labeled for NKB/KP/CART (Fig. 2F).

CART-containing KP and NKB neurons, including the triple-labeled ones, also sent abundant projections to the InfS (Figs. 1F–J).

**Figure 3:**
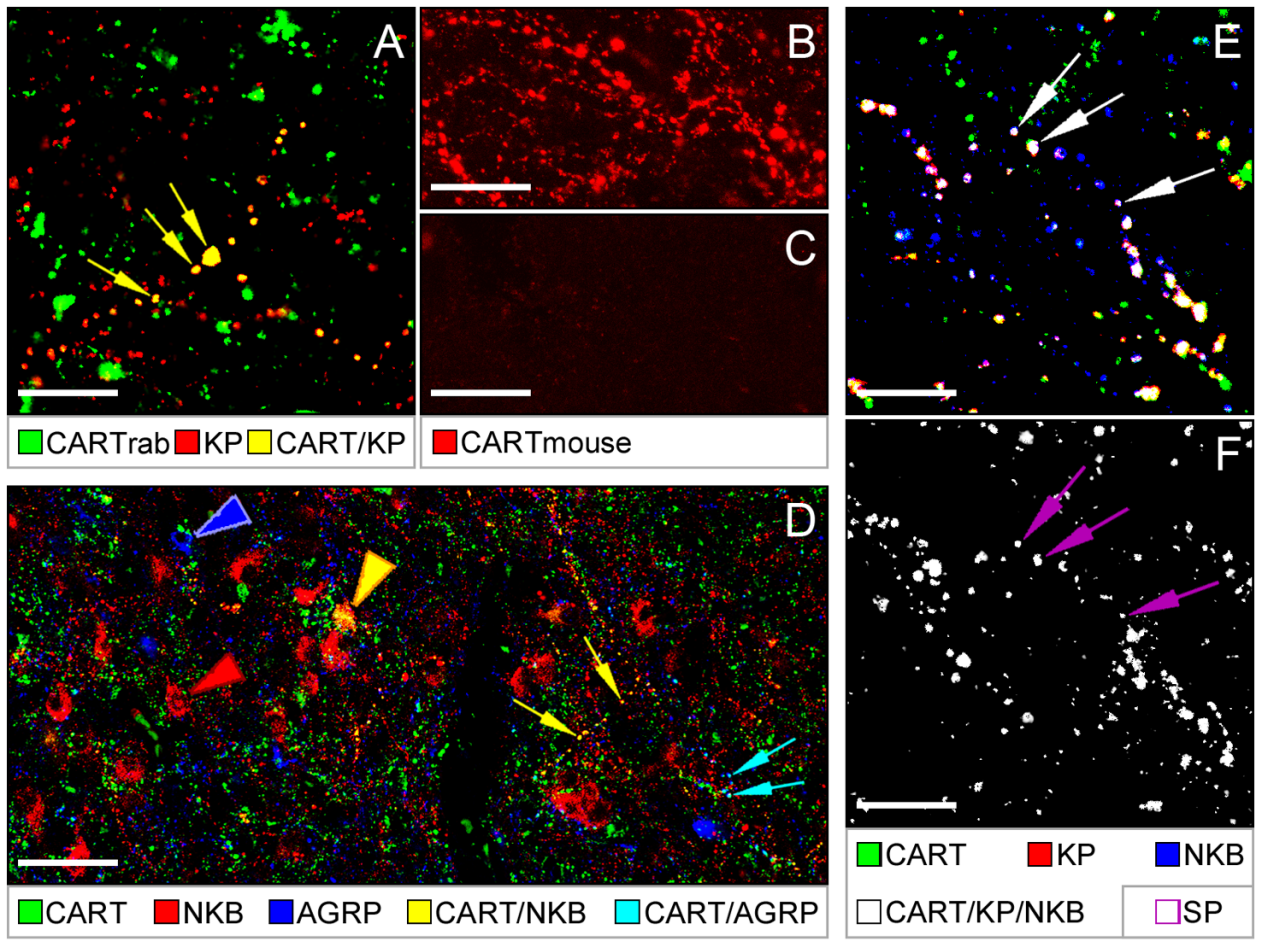
Further characterization of CART-IR KP and NKB neurons. Positive control experiments using a reference CART antiserum raised in rabbit (‘CART rab’) confirm the presence of CART immunoreactivity in KP neurons (**A**). Yellow arrows point to axon varicosities dual-labeled for CART (green) and KP (red). In further support of specificity, CART labeling of the Inf with the mouse CA61F4OP001 antibody (CART mouse; **B**) can be entirely eliminated by an overnight preabsorption of the antibody with 10^−4^ M human CART 54–102 peptide (**C**). Results of triple-labeling experiments in **D** indicate that CART-IR NKB fibers (yellow arrows) are distinct from CART fibers expressing the orexigenic peptide AGRP (turquoise arrows). Color-coded arrowheads point to labeled perikarya. Colocalization results from quadruple-immunolabeling studies (**E** and **F**) reveal that the CART-containing and the SP-containing subsets of KP and NKB neurons overlap. Arrows in **E** illustrate the projecting fibers of neurons triple-labeled for KP, NKB and CART. Arrows in **F** point to SP immunoreactivity in the same (quadruple-labeled) axon varicosities. Captions below the panels reveal color-coding. Scale bars = 20 µm in **A, B, C E,F** and 60 µm in **D**.

The CART co-labeling of the KP-IR neuronal elements could be reproduced in control experiments in which the mouse monoclonal CART antibody was replaced with the rabbit polyclonal CART antiserum (Fig. 3A). In addition, an overnight preabsorption of the mouse CART antibody with 10^−4^ M human CART 54–102 peptide eliminated all labeling from the human Inf (Figs. 3B and C).

### Experiment 3. NKB neurons with CART are distinct from CART neurons that express AGRP

CART-IR and AGRP-IR neurons of the human Inf are partly identical [24]. In this study, we have confirmed this colocalization phenomenon by showing that 5.9±1.1% of 1171 AGRP-IR axon varicosities from three individuals contained CART immunoreactivity. The quantitative analysis of 2417 CART-IR axon varicosities revealed NKB in 6.2±1.6% and AGRP in 2.9±0.7% of CART-IR fibers, without identifying a single case of overlap between AGRP/CART and NKB/CART fibers. These studies have established that CART-IR AGRP neurons and their fiber projections are distinct from the CART-IR NKB elements (Fig. 3D).

### Experiment 4. Subsets of CART-IR KP and NKB fibers also contain SP

Quadruple-immunofluorescent studies revealed cases when KP-IR and/or NKB-IR neuronal profiles co-contained SP with CART. The existence of quadruple-labeled axons indicated that the CART-containing and the SP-containing populations of KP and NKB neurons partly overlap (Figs. 3E and F).

### Experiment 5. CART-IR KP and NKB neurons innervate peptidergic cells, including GnRH neurons

**Figure 4:**
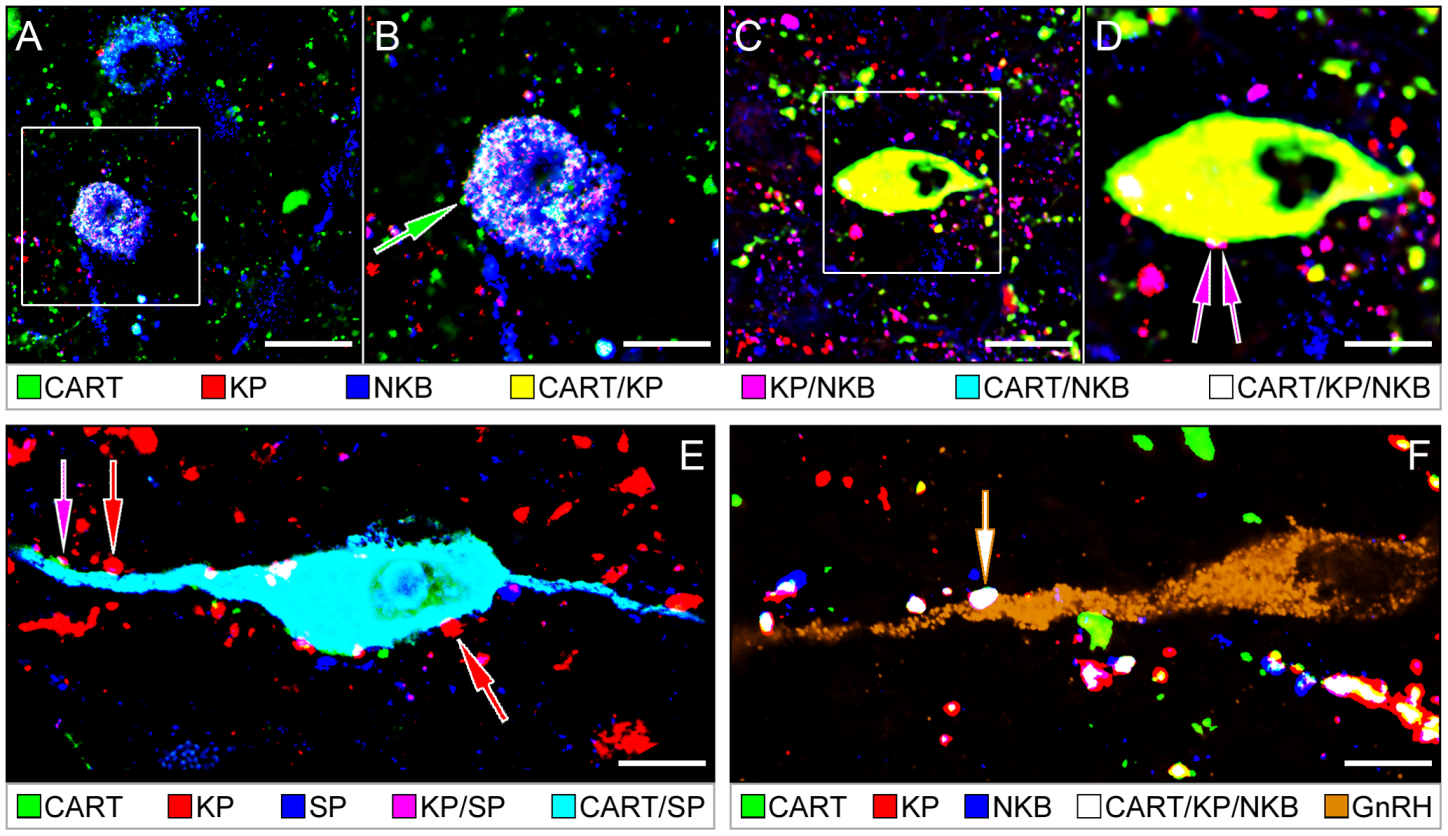
Neuronal interactions involving cocaine- and amphetamine-regulated transcript (CART)-containing kisspeptin (KP), neurokinin B (NKB) and substance P (SP) neurons in the infundibular nucleus (Inf). Neuropeptides exhibit variable colocalization patterns in neuronal cell bodies and fibers of the Inf (see color-coded captions). Single-, double- and triple-labeled peptidergic axons establish appositions onto other peptidergic neurons. Such appositions often involve CART-IR neuronal elements. The CART/KP/NKB triple-labeled cell body in **A** and **B** receives an axo-somatic afferent contact from a single-labeled CART-IR axon (green arrow in **B**). The CART/KP double-labeled cell body in **C** and **D** receives an afferent contact from a KP/NKB double-IR axon (purple arrows in **D**). A CART/SP double-IR neuron in **E** is innervated by KP-IR (red arrows) and KP/SP double-IR (purple arrow) axons. The dendrite of a GnRH neuron (brown pseudocolor) in **F** receives innervation from a CART/KP/NKB-IR triple-phenotype axon (white arrow). Scale bars = 20 µm in **A**, **C**, 12 µm in **E** and 10 µm in **B**, **D** and **F**.

CART-IR cell bodies and fibers were involved in neuronal contacts between the different types of peptidergic neurons expressing KP, NKB, CART and SP (Figs. 4A–E). In quadruple-immunolabeling experiments, CART was occasionally revealed within KP-IR and NKB-IR axons forming appositions to GnRH neurons. Some of these rarely encountered afferent inputs were triple-labeled for KP, NKB and CART (Fig. 4F).

## Discussion

This study provides morphological evidence for the presence of CART neuropeptide in 47.9±6.6% of the KP-IR and 30.0±4.9% of the NKB-IR perikarya and in 17.0±2.3% of the KP-IR and 6.2±2.0% of the NKB-IR axon varicosities in the Inf of postmenopausal women. The CART-IR KP and NKB fibers often contained SP as well, while being distinct from CART fibers co-containing the orexigenic peptide AGRP. Axon projections of the CART-IR KP and NKB neurons innervated the InfS and also established afferent contacts onto various peptidergic neurons of the mediobasal hypothalamus, including GnRH-IR cells.

### Species differences in the neuropeptide complement of mediobasal hypothalamic KP and NKB neurons

While human KP and NKB neurons contained CART in the present study, the same colocalization phenomenon has not been noticed in rodents in a recent study which showed confocal microscopic evidence for CART-IR afferents to KP neurons of rats [21]. Thus, it occurs that CART coexpression represents a further species-specific feature of mediobasal hypothalamic KP and NKB neurons, in addition to other neurochemical properties that differ between the human and laboratory animal species. Notably, unlike in rodents [2] and sheep [1], KP and NKB neurons of the human infundibular region and their axons rarely express detectable levels of dynorphin immunoreactivity [19]. On the other hand, while KP and NKB neurons in the human often exhibit SP immunoreactivity [20], similar colocalization has not been reported in the ARC of laboratory rodents. We note that in the present study we provide quadruple-immunofluorescent evidence for an overlap between the SP-expressing and the CART-expressing subsets of human KP and NKB neurons, suggesting that these neuronal populations are partly identical. Overall, the above and possible further species differences suggest that the co-transmitters and modulators that KP and NKB neurons use for interneuronal communication may largely differ among species.

### Functional significance of CART in human KP and NKB neurons

CART has been implicated in a variety of homeostatic and neuroendocrine functions, including the regulation of body weight, reward and thermogenesis [36]. CART is colocalized with several orexigenic and anorexigenic hypothalamic neuropeptides. It is present in melanin-concentrating hormone-IR neurons in the lateral hypothalamus of both rats and humans [24], [35] which stimulate food intake [37]. In rodents, CART is also colocalized with the anorexigenic POMC neuronal system of the ARC [22] and food restriction decreases CART mRNA expression in these cells [21]. The effects of CART on food intake largely depend on its site of action. Orexigenic effects were observed following intrahypothalamic injections into the ventromedial nucleus or the ARC [38], [39]. In contrast, CART can decrease [39]–[41], and conversely, CART antibodies increase food intake when administered intracerebroventricularly [40], [41]. While CART is synthesized by POMC neurons in the mediobasal hypothalamus in rodents [22], somewhat unexpectedly, it was found that the analogous POMC neurons of the monkey do not contain CART [23]. Moreover, anatomical evidence from the human indicates that in primates, CART is synthesized by the orexigenic AGRP/NPY neuronal system [24]. Results of our present study confirm this colocalization phenomenon and also provide evidence that AGRP/NPY/CART neurons of the human are distinct from CART neurons that synthesize NKB and/or KP.

In addition to playing a role in metabolic regulation, CART has also been implicated in reproductive functions, and thus, suggested to contribute to the inhibition of fertility under negative metabolic conditions [21]. CART fibers establish close contacts with GnRH neurons in rats and Siberian hamsters [21], [42], [43]. While these afferents may arise from multiple sources, over 80% of the afferent contacts in rats contain α-MSH immunoreactivity [21], indicating their origin in the ARC where the two peptides are co-synthesized [22]. Besides the ARC, the dorsomedial hypothalamus, the ventral premammillary nucleus and the anteroventral periventricular nucleus of the rat serve as further sources for CART fiber projections to the preoptic regions populated by GnRH neurons [43]. Fifteen [44] and 75% [21] of GnRH neurons in mice and rats, respectively, give excitatory responses to CART. Caloric restriction decreases CART mRNA expression and the number of CART-IR neurons in the anteroventral periventricular nucleus and the ARC [21], [41], suggesting that the excitatory CART input to GnRH neurons from these cell populations is decreased during negative energy balance. Leptin receptor containing neurons including CART cells in the ventral premamillary nucleus seem to be particularly important players in the metabolic suppression of reproduction [45].

CART neurons are also capable of modulating GnRH neuronal functions indirectly. CART-IR fibers innervate KP neurons of the ARC [21] and CART exerts excitatory actions on these cells [21]. It is important to emphasize that although such excitatory actions of CART on both GnRH and KP neurons are well established in rodents, conclusions of these experiments should only be applied to primates with great caution.

Similarly to KP, NKB and SP, CART may either act on autoreceptors or on postsynaptic peptide receptors to modulate GnRH and/or KP neuronal functions. CART signaling was proposed to involve G protein coupled receptors which remain to be identified. CART is localized to dense-core vesicles in the central nervous system [46]. High-resolution neuromorphological approaches will be required to clarify whether KP, NKB, SP and CART are localized to the same or to distinct dense-core vesicle populations. In either case, neuropeptides co-contained in the same axon varicosity are likely to be secreted simultaneously upon stimulation to act on the somato-dendritic and/or axonal compartments of other neurons in the network, including GnRH cells. While some functional redundancy is possible in the information carried by the co-released neuropeptides, it also seems likely that the absolute and relative amounts of co-secreted peptides are critically important in the functions associated with these cells, including negative sex steroid feedback [1], [7] and the regulation of GnRH/LH pulsatility [2]–[4]. Therefore, it will be crucial to analyze and understand the mechanisms regulating neuropeptide kinetics not only in cell bodies but also in axon varicosities and terminals.

### Neuropeptide colocalization patterns in perikarya vs. fibers

Earlier colocalization studies of the KNDy peptides focused on neuronal cell bodies/e.g.[1], [2]/with little attempt to provide quantitative estimates about the extent of neuropeptide coexpression in axon varicosities or terminals from which secretion is most likely to take place. In our present study, we have developed an approach to determine the degrees of neuropeptide colocalization separately in perikarya and in axon varicosities.

Comparative analysis of CART immunoreactivity in the different subcellular compartments revealed that CART occurs less frequently in axon varicosities than in cell bodies of KP-IR and NKB-IR neurons. This phenomenon suggests that only a fraction of CART-synthesizing KP and NKB neurons transport CART into their axon varicosities under basal conditions in the postmenopausal human model. It requires clarification and studies of additional endocrine conditions to determine whether and how the coexpression of CART and other neuropeptides vary in axon varicosities as an affect of the reproductive status. Of note, while an earlier study by Menyhért and colleagues revealed CART immunoreactivity in 35.7±2.2% of NPY-IR (AGRP) neurons of the Inf [24], in experiment 3 we only found CART signal in 5.9±1.1% of AGRP-IR axon varicosities, suggesting also that the limited transport of CART to axon varicosities may also characterize the AGRP/NPY neuronal system.

The relatively low degree of neuropeptide colocalization in axon varicosities is not a unique feature of CART. We also found that the extent of KP coexpression with NKB was significantly lower in fibers than in cell bodies. In our present study, the incidences of KP-IR perikarya with NKB (78.4±2.9%) and NKB-IR perikarya with KP (66.5±5.1%) were similarly high as in earlier colocalization experiments [26] on postmenopausal women (83.7±3.7% and 71.3±5.9%, respectively). In contrast, only 54.9±5.4% of KP-IR and 23.1±9.0% of NKB-IR axon varicosities expressed immunoreactivity for the other neuropeptide. This moderate KP/NKB coexpression in fibers is also in accordance with our earlier observations that single-labeled KP-IR and NKB-IR axons often occur in the Inf [25], [28] and that the majority of KP-IR and NKB-IR juxtapositions to GnRH neurons are also single-labeled [25]. The low degree of neuropeptide colocalization in axons has several possible explanations. First, it can not be entirely ruled out that low neuropeptide levels remained undetected in the single-labeled fibers. Second, not all of the immunolabeled KP, NKB and CART fibers are necessarily of local origin; KP, NKB and CART axons may innervate the Inf from other hypothalamic and extrahypothalamic regions where the peptides are not cosynthesized. Finally, single-labeling in most instances is likely to result from the disproportional KP and NKB synthesis, transport, degradation and release. It is known that the endocrine status can regulate the level of KP and NKB differentially; in men, the ratio of NKB-IR perikarya that also contain KP increases considerably with age [27]. The axons of these neurons also exhibit a sex-specific colocalization pattern of the two neuropeptides; KP and NKB were colocalized in 25–30% of KP-IR and NKB-IR afferents to GnRH neurons in postmenopausal women, but only in 8–10% of KP-IR and NKB-IR afferents in men above 50 years [25]. Future studies will need to address how the colocalization pattern of neuropeptides in axon projections of KP and NKB neurons affects the physiology of these cells.

Overall, the relatively low level of neuropeptide colocalization we observed in axon varicosities suggests that neurons capable of neuropeptide co-synthesis do not necessarily have all neuropeptides available for axonal release under all physiological conditions. The differential processing and transport of co-synthesized neuropeptides may thus have a robust impact on functions regulated by these neurons, in addition to the most intensely studied transcriptional regulation. We note that most of the previous studies on rodents tended to analyze the colocalization of KNDy peptides in cell bodies, without paying much attention to the same colocalization phenomena in neuronal fibers. It thus remains possible that rodent KNDy neurons also exhibit lower neuropeptide colocalization in axons than in perikarya, as it has been shown recently by True et al. These authors found nearly complete overlap between KP and NKB cell bodies in the mouse ARC, while reporting that only 39% of KP fibers overlapped with NKB staining, and 43% of NKB fibers overlapped with KP fibers in the median eminence [47].

In summary, anatomical evidence in this study suggests that CART plays a co-transmitter/neuromodulator role in functions regulated by human hypothalamic KP and NKB neurons. Target cells, receptor sites and physiological effects of intrinsic CART in the efferent communication of KP and NKB neurons require clarification.
